# COMPARATIVE ANALYSIS OF LONELINESS AND HEALTH: EFFECTS ACROSS DEFAMILIZATION LEVELS

**DOI:** 10.1093/geroni/igae098.1537

**Published:** 2024-12-31

**Authors:** Ariel Azar

**Affiliations:** Purdue University, West Lafayette, Indiana, United States

## Abstract

Although a significant amount of research has addressed the negative relationships of loneliness and social isolation with health in old age, the mechanisms linking them to health outcomes are not fully understood. While some argue that health behaviors may account for these associations, previous research has not adequately contextualized the relationships between loneliness, social isolation, and health. Given the variations in these relationships across different contexts, it is imperative to understand how institutional contexts moderate the detrimental effects of both loneliness and social isolation. This comparative study focuses on European countries and the U.S., utilizing SHARE data for 20+ European countries, ELSA data for the UK, and both HRS and NSHAP data for the U.S. It explores how the relationships between loneliness, social isolation, and health vary across institutional exposure to defamilization policies during the life course. These policies aim to promote women’s autonomy and reduce the caregiving burden traditionally placed on them. By leveraging panel data, this paper employs a causal framework to estimate how the effects of loneliness and social isolation on health vary for individuals experiencing different institutional contexts. This research introduces a novel approach to understanding the moderating role of the welfare state, viewing it not only as a resource-transferring institution but also as a facilitator of social networks.

